# New complementary alternatives in third molar autotransplantation: A systematic review

**DOI:** 10.4317/medoral.26233

**Published:** 2023-10-12

**Authors:** Juan Pablo Aravena-Salazar, Gustavo Matus-Miranda, Jessika Dethlefs-Canto, Sven Eric Niklander

**Affiliations:** 1 ORCID: 0000-0002-1089-8446. Unit of Oral and Maxillofacial Surgery, Faculty of Dentistry of Universidad Andres Bello, Viña del Mar, Chile; 2 ORCID: 0000-0003-0966-7576. Unit of Oral and Maxillofacial Surgery, Faculty of Dentistry of Universidad Andres Bello, Viña del Mar, Chile; 3 ORCID: 0009-0000-9624-0225. Unit of Oral and Maxillofacial Surgery, Faculty of Dentistry of Universidad Andres Bello, Viña del Mar, Chile; 4 ORCID: 0000-0003-1858-3091. Unit of Oral Pathology and Medicine, Faculty of Dentistry of Universidad Andres Bello, VinÞa del Mar, Chile

## Abstract

**Background:**

Dental autotransplantation (DAT) is defined as the replacement or direct transfer of an impacted, semi-impacted or erupted tooth to a donor site, either to a post-extraction socket or to a surgically created socket within the same individual. The use of new technological advances, such as 3-D dental models based on computer-aided design, among others, have been reported to improve the success rate of DAT. Therefore, we aimed to perform a systematic review to explore the possible benefits that the use of these innovative techniques can provide when applied to DAT.

**Material and Methods:**

The literature search was conducted in PubMed, Scopus, and Web of Science databases following the PRISMA guidelines. The research question was: "Are computerized technological advancements a useful tool for improving the success of third molar autotransplantation technique?

**Results:**

The initial literature search identified 195 articles, of which only 11 were included for qualitative analysis. All studies used 3D dental models based on computer-aided design data. Surgical guides and stereolithographic models were used by 4 and 1 study respectively. A total of 91 transplanted teeth were evaluated, out of which only 88 were considered within the parameters of clinical success (96.7%). Only 7 out of the 11 articles reported the specific autotransplanted tooth, being mandibular third molars the most prevalent autotransplanted teeth.

**Conclusions:**

Although the application of new technologies for DAT increases the success rate of this technique, further primary studies are still needed to address long-term teeth survival rates and complications. The cost and availability to implement the integration of these techniques to DAT may be a variable to consider, as this can be a limitation for some patients or for low-income countries.

** Key words:**Autotransplantation, third molars, digital planning.

## Introduction

A rarely used but recognized technique to restore the loss of a tooth is dental autotransplantation (DAT). DAT is defined as the replacement or direct transfer of an impacted, semi-impacted or erupted tooth to a donor site, either to a post-extraction socket or to a surgically created socket within the same individual (1-6). The term DAT was introduced in 1728 by Fauchard *et al*. and first applied by Miller *et al*. in 1950 (2,3,7). Since then, it has been reported as a useful indication for teeth replacement due to different causes, including: extensive non-restorable caries, trauma, periodontal disease, endodontic failures, agenesis, among many others (2,6,8,9). It might be contraindicated due to medical conditions, poor oral hygiene, unsuiTable donor tooth, or inadequate remaining bone (10).

DAT not only allows for the preservation of aesthetics and function, but also exhibits physiological characteristics compatible with a normal tooth. The best example for that is that auto-transplanted teeth are eligible for orthodontic movement, as the periodontium is maintained in optimal conditions, presenting a favorable long-term prognosis (2,10,11). While this treatment demonstrates favorable outcomes in mature teeth with complete root formation and when associated with prior endodontic treatment or performed within 2 weeks after the procedure (9,10,12,13), the most opportune time to consider DAT is in young patients with root formation ranging from 50% to 75%. This allows for a high probability of revascularization, apexification, and integrity of the periodontal ligament, while also contributing to the neoformation of bone tissue avoiding complications such as root resorption or ankylosis (2,3,6,10,14).

 The replacement of teeth through implant-supported rehabilitations is a widely accepted and first-choice therapeutic approach (15). However, DAT presents significant advantages over dental implants, including proprioception during function, a vital periodontium, preservation of alveolar bone volume, preservation of the papilla and a reduced cost (6,7).

Currently, new technologies are being applied to DAT of third molars, allowing the development of surgical guides and 3D prototypes, which facilitate and improve surgical management (1-3,5,7,10,16). Therefore, the aim of this study was to perform a systematic review to explore the possible benefits that the use of these innovative techniques can provide when applied to DAT of third molars.

## Material and Methods

- Study design

This research work was guided by the PRISMA (Preferred Reporting Items for Systematic Reviews and Meta-Analyses) extension protocols (17). The research question was: "Are computerized technological advancements a useful tool for improving the success of third molar autotransplantation technique?"

- Eligibility

The included articles were those available in full text, in English language, and reported the use of innovative techniques in the planning and execution of third molar autotransplantation, including: computerized tomography, virtual softwares, 3D surgical guides, and 3D printing. Only cohort studies, clinical studies (randomized or non-randomized), prospective and comparative studies, retrospective studies, case reports, and case series were considered, without limitations on their publication date. Animal studies, narrative reviews, systematic reviews, *in vitro* studies, and studies unrelated to the research question were excluded.

- Sources of information

The authors conducted an independent search in PubMed, Scopus, and Web of Science databases between the December 17, 2022 and January 22, 2023.

- Search Strategy

The following search algorithms were used: (Dental autotransplantation AND Surgery AND Technology), (Autotransplantation AND Digital planning AND Third molars), (Third molars AND Guided autotransplantation), (Third molars AND 3D dental replica AND Autotransplantation), (3D dental replica AND Autotransplantation), (Digital planning AND Autotransplantation AND Dentistry). Additionally, articles within the references of each evaluated manuscript that aligned with the objectives of this study were also considered.

- Article selection

**Figure 1:**
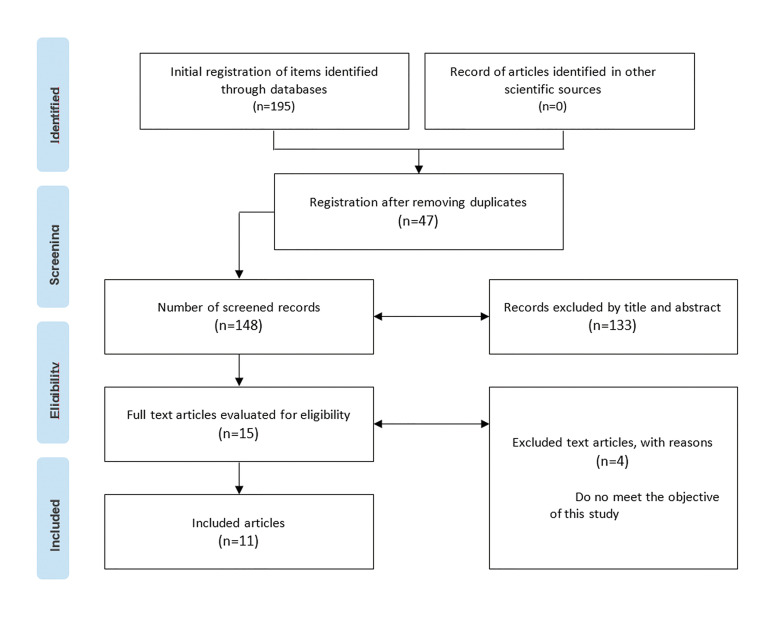
PRISMA diagram.

The selection of articles was independently performed by 2 reviewers (JPA and GM). The reference manager Mendeley was used for exporting the primary data. Titles and abstracts were analyzed to identify articles eligible for a comprehensive review (Fig. 1). Disagreements were resolved through discussion or involvement of a third reviewer.

- Data extraction

Data extracted from the included studies were tabulated into Microsoft Excel and presented in a detailed manner in the form of Tables and Figures.

- Risk of bias

No bias analysis was conducted due to the lack of methodological quality and low scientific evidence of the included studies. Instead, a critical assessment of study quality, identifying limitations, was performed.

## Results

The literature search identified 195 articles. Duplicates across databases were removed, resulting in 148 publications, which underwent a detailed review of titles and abstracts. One hundred and thirty-three studies did not align with the objectives of this study and were subsequently excluded. A total of 15 potential manuscripts were chosen for full-text evaluation. Among them, 4 were excluded for specific reasons, leaving only 11 articles included for qualitative analysis (Fig. 1).

The included studies were published between the years 2017 and 2021. Three were from China, 3 from Spain, 2 from Korea, 1 from the Netherlands, 1 from Turkey, and 1 from Japan. Out of all the included studies, 6 were case reports, 3 were case series, and 2 were retrospective studies. The total number of patients was 89, with 3 of them not completing their follow-up. The female gender (*n*=51) was reported in a higher percentage (57%) compared to the male gender (*n*=38), with an age average ranging from 17 to 64 years (Table 1).

A total of 91 transplanted teeth were evaluated, out of which only 88 were considered within the parameters of clinical success (a success rate of 96.7%). Only 7 out of the 11 articles reported the specific autotransplanted tooth, being mandibular third molars the most prevalent autotransplanted teeth. The teeth to be replaced presented different conditions, including: no restoring possibilities, a history of severe infection, the need for coronal elongation, the need for apicoectomy, failed endodontic treatments, failed restorations, agenesis, early tooth loss, root fracture, and extensive caries, the latter being the most frequent condition (Table 2).

Regarding the third molars to be transplanted, 27% (*n*=3) of the studies reported teeth with three-quarters of root formation, 36% (*n*=4) with complete root formation, and the rest did not describe it (*n*=4). Out of the total articles, 73% (*n*=8) reported the need for post-surgical endodontic treatment, 18% (*n*=2) did not require it, and the rest did not describe it (*n*=1). While the most frequently reported anatomical disposition of the donor tooth was impacted, only 27% (*n*=3) of the articles considered this variable within their study.

Various technologies were used. All studies (*n*=11) focused mainly on the use of 3D dental models based on computer-aided design data over the use of surgical guides, which was present in only 36% (*n*=4), and stereolithographic models, with 9% (*n*=1). The last being considered as a complement to the 3D dental modeling technique. In addition to conventional 2D radiographs, more advanced 3D imaging studies were also employed as an adjunct, and CBCT was found to be the most effective in terms of planning and accuracy.

**Table 1:**
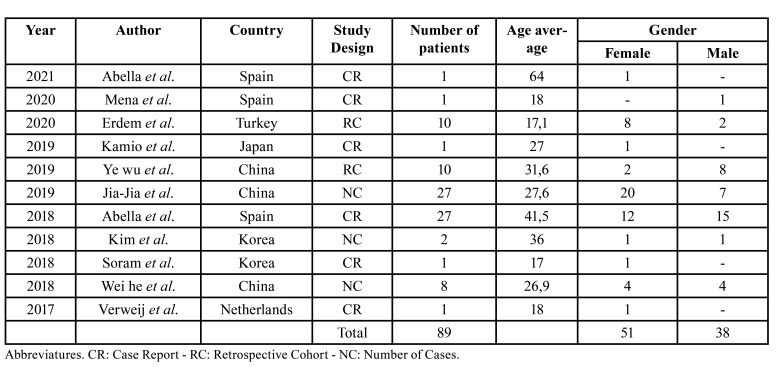
Demographics of the included studies.

**Table 2:**
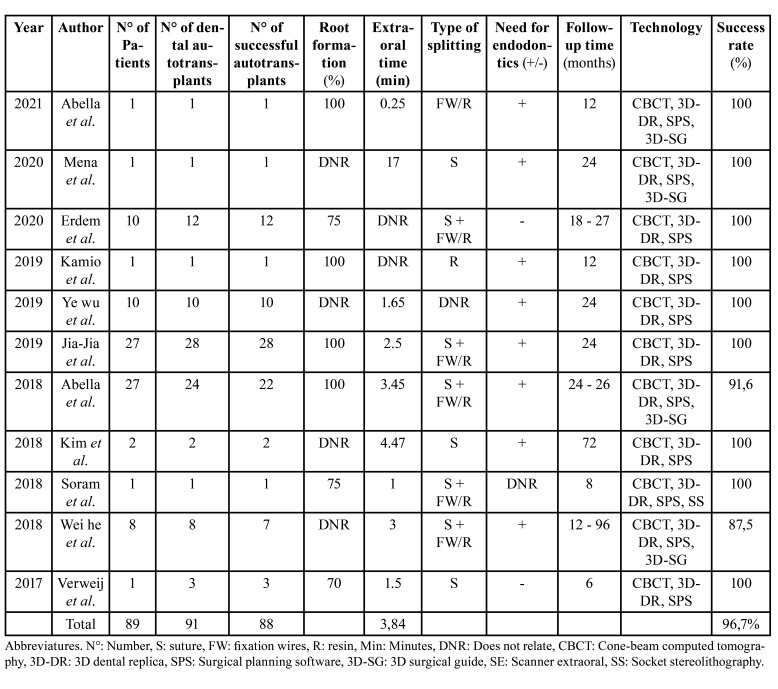
Studied variables.

Regarding the use of medication, only 73% of the articles (*n*=8) mentioned a pharmacological approach both pre- and post-operatively. All authors indicated the use of splints (archwires, composite resin, and sutures). The removal of these varied within a range of 7 to 90 days. Similarly, the follow-up time among the articles was heterogeneous, ranging from 6 to 96 months.

## Discussion

Compared to other treatments, autotransplantation of third molars presents physiological properties that position it as an effective treatment alternative. In comparison to dental implants, fixed or removable prostheses, DAT allows for the preservation of proprioception, a vital periodontium, greater occlusal resistance, pulpal revascularization, increased preservation of bone volume, dentoalveolar development, improved aesthetics, and maintenance of interdental papilla. When combined with the use of the aforementioned technologies, autotransplantation can ensure a higher likelihood of clinical success with a favorable postoperative outcome (1,2,4-7,18,19).

Despite its advantages, DAT is not a frequently used technique. A retrospective study analyzing autotransplants performed over a 20-year period in Japan reported an average of 1.4 patients undergoing this treatment, highlighting the low frequency of DAT indication, probably due to both patients and clinicians concerns about treatment success (18,20).

The literature indicates the use of conventional unguided DAT techniques with variable success rates (59%-81%). Variables considered for conventional DAT are: tooth type, degree of root formation, patient gender, alveolar socket type (recent extraction or surgically created), surgical trauma, alveolar bone quality, extra-oral time of the donor tooth, adaptation attempts, root morphology, stabilization methods, operator skill, periodontal ligament integrity and good adaptation of the surrounding tissues (5-7,21). Integrity of the periodontal ligament and adaptation of the surrounding tissues are considered as the most important factors for achieving clinical success (7).

In order to increase the success rate of DAT, technological advances have been incorporated to this procedure. Since the introduction of computer-aided prototypes (dental replicas, life-sized recipient alveolar bone, surgical guides, splints, and 3D simulations), along with the use of biocompatible and biodegradable materials and guided cone-beam computed tomography (CBCT), better control has been achieved over the aforementioned challenges (16,22). Ye Wu *et al*. reported the use of 3D dental replicas as a valuable option for replacing the donor tooth and determining if the actual alveolus is suiTable as a recipient site, thereby minimizing the extra-oral time of the transplanted tooth (0-4 min) (7). This approach avoids collateral damage to the Hertwig's epithelial sheath and the periodontal ligament due to adaptation attempts (3,7), as the viability of the periodontal ligament decreases after 18 minutes outside its physiological alveolus (2,4). Verweij *et al*. reported a decrease in surgical time to less than 30 minutes when using dental replicas, even when performed by not experienced operators (10). This corresponds with the results of another study, in which the authors found a reduction in intraoperative time between 10-45 minutes when comparing conventional DAT with DAT supplemented a clinical imaging-based software (CBCT) for 3D printing of a dental model (7).

One of the most commonly reported complications of DAT is root resorption, particularly caused by traumatic extraction or excessive pressure during the placement of the donor tooth in the recipient site (1,8). This complication can be controlled by using surgical guides, which allows for three-dimensional positioning of the dental replica and subsequent placement of the donor tooth, thus avoiding further damage to the root (1).

The use of surgical models has been shown as an important tool to increase the success of DAT, and the accuracy of the models is crucial for it. Several factors have to be considered in this respect, such as CBCT data, material shrinkage (resin, starch, chrome-cobalt), minimum layer thickness, among others (7,10). There is no standardized definition regarding the minimum discrepancies between the 3D model and the donor teeth (7), although several studies have reported that 0.25 mm is clinically accepTable (7,8,23). Other authors have reported a mean absolute error of 0.291 mm compared to real teeth, yet still achieved good results (5,7,16). Another factor described as relevant in DAT is the age of the patient. A higher success rate has been reported in patients under 40 years, and a significant decrease in success rate in patients aged 55-69 years (1,3,7,24).

During the planning phase, the cervical fit between the donor tooth and alveolar bone is considered a decisive variable, as its proper sealing allows for better healing tendencies, minimizing bacterial invasion and reducing the risk of infection. Poor bucco palatal/lingual positioning can lead to resorption of the alveolar crest, contributing to treatment failure (2,4). For the preparation of the recipient site, it is suggested that neither the depth nor the width should be excessively large, recommending 2 mm and 1 mm deeper and wider than the donor tooth respectively (4). The use of 3D simulators, that allow correlating anatomical/volumetric discrepancies between the donor tooth and recipient site are highly recommended (5).

The stability of the transplanted tooth is crucial, and studies support this, reporting an increase in the healing rate in cases with good initial stability (3). The literature on donor tooth fixation is heterogeneous. Sutures are commonly the first alternatives, with a total time use ranging from 7 to 90 days. In cases of failure, wires and composite fixation are recommended (16,18). The use of flexible splints for a period of 7 to 10 days has been recently recommended, as the incidence of complications is directly related to the revascularization of the transplanted tooth. Flexible splints allow functional movement, inducing cellular activity in the periodontal ligament promoting bone regeneration (3,6,25).

Although DAT presents morbidity to both soft and hard tissues, the average post-procedural pain experience (based on the visual analog scale or VAS), has been reported of 1.25 ± 0.75, with a VAS score of 0 on the seventh day (4). In terms of pharmacological management, none of the analyzed articles explicitly suggested an ideal approach, but antibiotic treatment, anti-inflammatory drugs, and analgesics are commonly used alternatives (1-3). Regarding postoperative care, in addition to the aforementioned measures, the use of a 0.12% chlorhexidine antiseptic mouthwash is recommended (3). As for costs, only one study provided information on the cost of using chrome-cobalt dental replicas produced by experienced 3D laboratories, which was around 30 USD. Considering its accessibility and straightforward workflow, DAT can be regarded as a cost-effective technique (23).

Despite the demonstrated success rate, it is necessary to consider the methodology of the included studies as a limitation and potential bias in the results of this review. In fact, 73% (*n*=8) of the studies corresponded to case series or case reports, while the remainder are retrospective cohort studies. Therefore, it is important to encourage the scientific community to further investigate this topic with a larger study population and a methodology that effectively validates and compares the use and safety of autotransplantation as a treatment option.

## Conclusions

Although the application of new technologies for DAT increases the success rate of this technique, further primary studies are still needed to address long-term teeth survival rates and complications. The cost and availability to implement the integration of these techniques to DAT may be a variable to consider, as this can be a limitation for some patients or for low-income countries.
